# Enhancing Traumatic Stress Recovery Through Nonattachment Principles: A Scoping Review

**DOI:** 10.3390/jpm15120614

**Published:** 2025-12-09

**Authors:** Lindsay Tremblay, William Van Gordon, James Elander

**Affiliations:** School of Psychology, University of Derby, Derby DE22 1GB, UK; w.vangordon@derby.ac.uk (W.V.G.); j.elander@derby.ac.uk (J.E.)

**Keywords:** nonattachment, trauma, mindfulness, mindfulness-based interventions, post-traumatic stress, scoping review

## Abstract

**Background**: Nonattachment is an important component of Mindfulness-Based Interventions (MBIs), including its application within post-traumatic stress (PTS) contexts. However, within trauma contexts, there is limited understanding of the role and effectiveness of MBIs that integrate nonattachment. **Objective**: This study aimed to identify and evaluate evidence regarding the effectiveness of MBIs with nonattachment elements used with PTS populations. **Methods**: This review followed the PRISMA scoping guideline framework with searches conducted using Science Direct, PsycINFO, PubMed, and Google Scholar for peer-reviewed studies of MBIs with nonattachment principles or practices, and outcome measures related to PTS. The final search was conducted in January 2024, with no date restrictions for eligible studies. **Results**: Fourteen studies met the inclusion criteria including 7 randomized controlled trials, 4 cohort studies, and 1 quasi-experimental, 1 cross-sectional, and 1 qualitative study. Individual study samples ranged from 9 to 209 participants (n = 913). All studies showed promising results for interventions integrating nonattachment applied to PTS populations, with the MBI outperforming control conditions in 6 of 7 RCTs, and all cohort studies showing significant improvements. Improvements included reductions in PTSD assessment scores, stress and anxiety, negative self-concept, disturbances in relationships, expressive suppression and rumination, and experiential avoidance, as well as increased acceptance and compassion. Various quality issues were identified such as a lack of or poorly defined randomization, blinding procedures, controls for confounding variables, and small sample sizes. MBIs integrating nonattachment that target physiological stabilization, coupled with participant input into intervention decisions, appear most promising. **Conclusions**: MBIs that incorporate nonattachment elements may offer meaningful support for individuals experiencing PTS, particularly by fostering more flexible and less self-fixated ways of relating to thoughts and emotions.

## 1. Introduction

The Substance Abuse and Mental Health Services Administration (SAMHSA) [1] asserts that trauma results from harmful event(s) experienced by an individual that have lasting adverse effects on well-being. This is consistent with the definition provided by van der Kolk [2], which incorporates three essential criteria put forth by SAMHSA; Trauma is an event (criterion a) that has overwhelmed the central nervous system (criterion b) whereby the story of trauma is not in the past, but a current imprint or manifestation of pain presently living inside someone (criterion c). This provides an important inclusion of the element of psychological and physiological response in acknowledging adverse effects on the nervous system. Such adverse effects may occur immediately or over time and have the potential to last for months or years, resulting in chronic symptoms such as hypervigilance, numbing, and avoidance, which differentiate trauma from short-term reactions to adverse events [3].

Trauma shares considerable overlap with the definition of Post-Traumatic Stress Disorder (PTSD), which is a psychological response to a significant traumatic event characterized by intense stress. While all traumatic events are stressful, not all stressful events are traumatic. Traumatic events involve a significant threat to life, bodily integrity, or safety, which can lead to post-traumatic stress symptoms and responses. While the Diagnostic and Statistical Manual of Mental Disorders [4] specifies the relevant criteria for diagnosis, such criteria do not encompass all forms of trauma and there is considerable research speaking to their inadequacy [5,6,7,8]. Indeed, people may experience a wide range of Post-Traumatic Stress (PTS) symptoms, while failing to meet criteria for diagnosis of PTSD [9].

It is generally agreed that not only the nature of the traumatic event should be considered, but also the individual’s experience of it [1]. This experiential aspect may be the element of traumatic stress response most conducive to any application of agency. That is, while we cannot always control or influence the events we experience in life, we may be able to influence how those events are experienced in our own bodies and minds, how we connect with them and how we attach to them. Frequently reported symptoms of PTS include a profound sense of helplessness, often in response to the experience of a significant loss of control [10]. Thus, an appropriate therapeutic response to PTS symptoms may involve targeting the response to the event, such as the need or drive to control, or the challenging nature of acceptance. Nonattachment, which entails an absence of attempts to control [11], may therefore be an appropriate avenue to explore for treatment of PTS symptoms. Nonattachment cultivates engagement with experiences as they are, and a release of the need for them to be otherwise [12]. Nonattachment also entails a flexible relationship with reality, which may help alleviate some of the suffering associated with PTS symptoms through acceptance, surrender, and a release of the need to control [13].

Notwithstanding conceptual overlap, recent work situates nonattachment as conceptually distinct from mindfulness, acceptance, and decentering [14,15,16]. Mindfulness emphasizes nonjudgmental present-moment awareness [14,15]. Acceptance refers to a willingness to experience events as they are without avoidance [16]. Decentering denotes observing thoughts and feelings as transient mental events [16]. Nonattachment extends these by incorporating *release of identification* with experience and freedom from outcome dependence. One may be mindful, accepting, or decentered, yet still attached to desired identities or results [17]. This broader relational stance helps dissolve habitual striving and self-fixation, both of which contribute to trauma-related suffering.

Despite its relevance, no literary reviews of MBIs for trauma have addressed nonattachment directly, often including it under the heading of mindfulness or acceptance instead. Consequently, the distinct mechanisms and empirical foundations of nonattachment remain unclear. There are past reviews of Mindfulness-Based Interventions (MBIs) for PTSD in which, for example, Taylor et al. [18] found significant effects in various populations suffering from psychological trauma, and Ramachandran et al. [19] found less pronounced effects among nurses suffering from PTSD. However, a specific focus on nonattachment within these MBI reviews is limited. Indeed, only 2 of the 80 studies comprising the two aforementioned reviews arguably featured interventions with a distinct nonattachment component, which is consistent with the general paucity of research on nonattachment in clinical settings.

Empirically, nonattachment has been examined using self-report tools such as the Nonattachment Scale [20] and the Nonattachment to Self-Scale [21], although few trauma-related studies have applied these measures directly. More commonly, nonattachment is inferred through related therapeutic mechanisms such as acceptance, nonjudgement, and letting go. An example of this can be seen in Acceptance and Commitment Therapy (ACT), a mindfulness-based form of therapy that is arguably a derivative of CBT [16], which avoids emphasis on altering specific cognitions or emotions (e.g., challenging or modifying assumptions to alter the downstream effect of catastrophizing or isolating as in CBT), and instead focuses on the context of the behaviors, known as de-coupling or decentring [16]. ACT seeks to alter the way one relates to internal experiences and reduce the amount of influence these experiences have on behaviour as a result [16]. An example of this is when negative self-perceptions are noticed as mere thoughts, not objective truths, and no longer prevent someone from engaging socially as they once may have. Interventions in this vein may focus on a shift away from the literal (e.g., ‘No one likes me’ is literally true and so I should isolate) to the non-literal (e.g., I’m having the thought ‘no one likes me’ which is coming from my own mind).

This reclassification of thoughts from ‘part of self’ to external, transient, and finite by-products of existence can also be understood through the lens of nonattachment and may account, in part, for its conflation with closely related constructs. However, nonattachment has roots in Eastern contemplative disciplines such as Buddhism and Hinduism [20]. It is generally defined as a flexible and balanced way of engaging with the world without attempting to control outcomes, or fixating on experiences [20,21]. Positioned affirmatively and in its refined form, nonattachment entails acceptance, letting go, deep presence, applying a universally interconnected self-schema, and cultivation of perceptual distance between both experiences, and one’s response to them [12]. As already noted, while nonattachment and decentring may share conceptual overlap, they are independent constructs [14]. For example, where an individual may be able to practice mindfulness and decentre from experiences, much like how ACT focuses away from the literal to the non-literal, they may still hold attachments to ideas about self and life in general [21].

Relinquishing ideas about self has been researched within the context of nonattachment-to-self (NTS) [21]. Here, ideas related to self as a separate or static construct existing independently of the rest of the world have the potential to create suffering due to beliefs that fulfilling the desires of self may yield happiness [22]. NTS speaks to a relationship with the concept of self without fixation or a need for things to be a certain way [21]. This links with the second element of SAMHSA’s [1] definition of trauma, which is not the traumatic (environmentally contingent) event, but the individual’s experience of it. Additionally, given that effective trauma interventions should acknowledge risk factors for PTS, nonattachment may offer value by virtue of its basis in the second element of trauma; the individual’s experience of it.

A growing body of research supports the many benefits of nonattachment, including greater wellbeing [23], increased self-esteem, empathy, and prosocial behaviours [13], improved relational harmony [24], improved wisdom, self-actualization, and self-transcendence [25], reduction in symptoms of anxiety and depression [26], and reduced suicidal ideation [27]. Although distinguishable from mindfulness, nonattachment is positively related [14]. Mindfulness is said to result from an open, nonjudging awareness toward events in one’s field of consciousness in real time [15], which supports research indicating that nonattachment may be a mechanism of mindfulness [25]. Additionally, MBIs have been shown to increase trait levels of nonattachment [28]. Using MBIs to increase levels of nonattachment, as measured by the nonattachment scale [20], has been shown to mitigate the severity of PTSD symptoms, sensitivity to anxiety, sensitivity to rejection, as well as improve empathic concern and personal distress [25]. Furthermore, one study showed that cultivation of nonattachment yielded benefits on broader psychological welfare but found that mindfulness alone may not be a significant factor in PTS symptom reduction [25]. This may be due to implicit negative biases related to nonattachment, or some general ambiguity related to what it really is [12].

Nonattachment and mindfulness may act as mediators for one another [25]. More specifically, mindfulness appears to nurture nonattachment, which in turn may permit a more inclusive engagement with experience, free from projections of self, fixations, or the need to control outcomes [25]. That is, MBIs that include nonattachment elements may have increased efficacy as a result, especially in PTS contexts where the experience of helplessness can be so difficult and thus a more accepting and flexible relationship with reality may be of benefit. This is particularly salient in the context of PTS due to the prevalence of dissociation and similar withdrawals from life as it is in the present moment [2]. Furthermore, given the uniqueness of external events required for the onset of PTS symptoms, understanding how nonattachment may influence response to environmental triggers may offer critical insights into ways to alleviate suffering.

Such a review is important given the shift toward greater integration of contemplative insight principles within mindfulness approaches as well as studies showing the combined effects of nonattachment and mindfulness for increasing perceptual distance from maladaptive thoughts, feelings, sensations, and memories [29]. To date, no literary reviews appear to map and evaluate studies of MBIs that explicitly integrate nonattachment principles or practices for individuals experiencing PTS. By delineating how nonattachment has been conceptualized, operationalized, and linked to outcomes, this review aims to clarify its theoretical position within second-generation mindfulness approaches and its potential role in trauma recovery.

## 2. Methods

A scoping review was chosen to map how nonattachment is conceptualized and operationalized within MBIs for PTS, to identify gaps in direct measurement, and to summarize the range and nature of existing evidence. The review followed the Preferred Reporting Items for Systematic Reviews and Meta-Analyses extension for Scoping Reviews (PRISMA-ScR) framework [30] (see Supplementary File S1 for the corresponding checklist). The review protocol was not preregistered, consistent with PRISMA-ScR guidance that registration is optional for scoping reviews [30].

A systematic search was conducted across four major academic databases selected for their disciplinary relevance and breadth of coverage in psychology, clinical and contemplative science: ScienceDirect, PsycINFO, PubMed, and Google Scholar. The final search was conducted in January 2024, with no date restrictions for eligible studies. The following Boolean search string was used in all databases, adapted as required for syntax compatibility: (“mindful” OR “meditation” OR “yoga” OR “nonattachment”) AND (“post-traumatic stress” OR “trauma” OR “PTSD”).

Search terms were applied to title, abstract, and keyword fields. Broader contemplative terms such as *yoga* and *meditation* were intentionally included to identify studies that might have implicitly incorporated nonattachment-related practices, as prior literature indicates the absence of standardized operational guidelines for cultivating nonattachment [12]. Reference lists of included studies and relevant citations were hand-searched to capture additional eligible articles not retrieved by the primary search.

### 2.1. Eligibility Criteria

Eligibility criteria were pre-defined according to PRISMA-ScR guidance to promote transparency and reproducibility. **Inclusion criteria:** 1. Peer-reviewed journal articles published in English. 2. Empirical intervention studies integrating nonattachment principles or practices, either explicitly (e.g., through mention or measurement of nonattachment) or implicitly (e.g., via contemplative techniques emphasizing letting go, acceptance, or reduced fixation). 3. Studies applying a mindfulness-based intervention (MBI) targeting outcomes related to psychological trauma, post-traumatic stress (PTS), or PTSD. 4. Quantitative, qualitative, or mixed-methods designs reporting measurable psychological outcomes. **Exclusion criteria:** 1. Conceptual papers, commentaries, reviews, or meta-analyses without new empirical data. 2. Interventions not grounded in mindfulness or contemplative principles (e.g., CBT, psychopharmacology, traditional psychotherapy). 3. Studies focused exclusively on physiological or somatic trauma without psychological outcomes.

### 2.2. Operationalization of Nonattachment

For the purposes of this scoping review, nonattachment was operationalized using a two-level framework developed during protocol planning. First, a measurement-based criterion was applied: studies were considered to operationalize nonattachment if they used a validated psychometric instrument designed to assess the construct (e.g., the Nonattachment Scale; Nonattachment-to-Self Scale).

Second, a conceptual-practice criterion was established to capture studies that did not measure nonattachment directly but *explicitly* referenced practices, psychoeducational content, or therapeutic intentions that align with contemporary definitions of nonattachment. To meet this criterion, studies had to articulate elements such as letting go, reduced identification with thoughts or emotions, decentring, impermanence-related teaching, equanimity, or non-fixation in a way that clearly connected these processes to the intervention’s mechanisms or aims. Generic mindfulness, ACT, Mindfulness Based Cognitive Therapy (MBCT), or Mindfulness Based Stress Reduction (MBSR) content was not considered sufficient unless nonattachment-relevant processes were explicitly mentioned by the authors.

These operational definitions were established a priori and applied consistently during screening and data charting to ensure conceptual clarity and prevent over-extension of the construct, given the historical ambiguity surrounding how nonattachment is applied within mindfulness-based interventions.

### 2.3. Screening and Data Extraction

Data were organized into table format to search for patterns across the dataset which may be relevant to understanding the effectiveness of nonattachment-based content or focus within existing MBIs. Reporting bias was addressed through an iterative and cumulative process of secondary and tertiary review of the articles selected for inclusion by the primary researcher, as well as secondary audit of the final selection by another member of the research team. Disagreements were resolved through discussion and consensus; no unresolved discrepancies remained.

Full-text screening followed the same independent verification procedure. Data were extracted into a structured template capturing study design and country of origin, participant population and sample size, intervention type and duration, explicit or inferred nonattachment components, outcome measures, and principal findings.

### 2.4. Study Quality

Although critical appraisal is not required for scoping reviews under PRISMA-ScR [30], this review conducted an optional quality assessment to strengthen interpretive confidence. Study quality and potential sources of bias were systematically appraised using the Standard Quality Assessment Criteria for Evaluating Primary Research Papers from a Variety of Fields (SQAC) [31]. The 14-item validated SQAC checklist was deemed to be an appropriate assessment tool given its focus on assessing methodological rigor and transparency of primary research across quantitative, qualitative, and mixed-methods studies. Each criterion (e.g., “Study design evident and appropriate?”, “Results reported in sufficient detail?”) was rated on a 3-point scale (2 = yes, 1 = partial, 0 = no), with non-applicable items excluded from scoring. For example, randomization and control-group criteria were excluded for qualitative studies, while qualitative reporting items (e.g., theoretical saturation, reflexivity statements) were excluded for quantitative studies. This ensured that studies were not penalized for methodological features inappropriate to their design. Using the following SQAC formula, individual study scores were converted to a percentage score: *Total Sum of Scores/Maximum Possible Score × 100.*

For each included article, both reviewers independently applied the SQAC criteria to evaluate clarity of research objectives, sampling methods, analytic transparency, and internal consistency of results. A structured quality-assessment table (available upon request) records each study’s individual item scores and overall quality percentage, allowing reproducibility of ratings.

Potential risk of bias was examined through the lens of SQAC domains, which encompass selection bias (sampling and recruitment), measurement bias (clarity of instruments and variables), and reporting bias (adequacy of data presentation and discussion of limitations). To enhance reliability and reduce subjective bias, the two reviewers conducted independent scoring, then compared results using consensus discussion. Inter-rater agreement was high (κ = 0.91). A single discrepancy occurred, for which the mean of the two ratings was adopted as the final score.

Scores were categorized as high quality (≥80%), moderate quality (60–79%), or low quality (<60%). Descriptive summaries of these categories are provided in the Results section to facilitate interpretation of study quality and confidence in the synthesized evidence, ensuring that study quality appraisal was conducted systematically, reproducibly, and in alignment with PRISMA-ScR recommendations on methodological transparency.

## 3. Results

**Figure 1:**
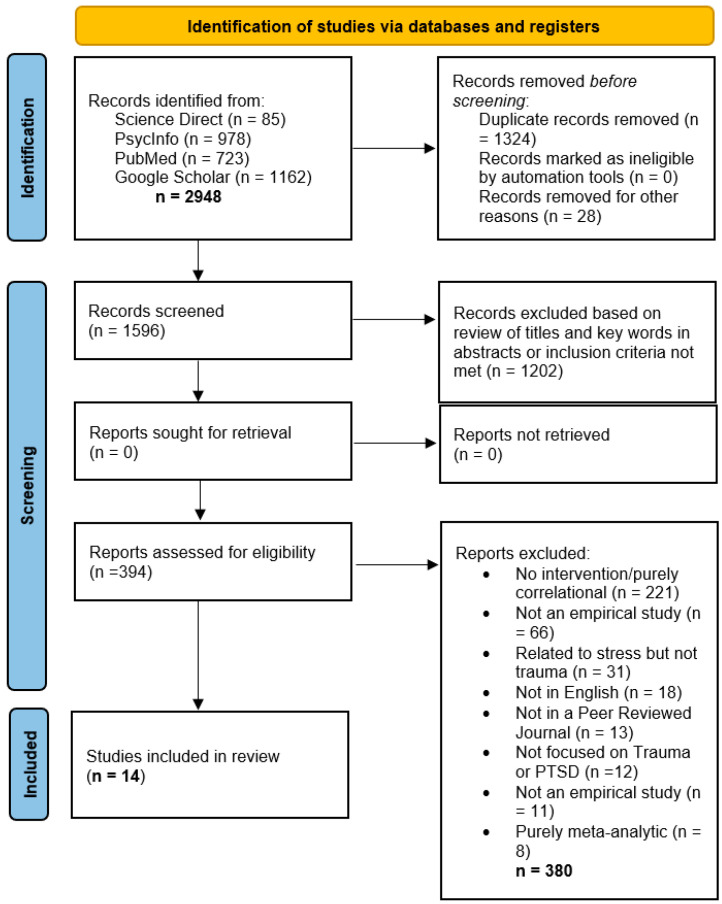
PRISMA flow diagram of the search process.

The initial search, summarized in Figure 1, yielded a total of 2948 publications between all four databases. Following the described screening process, fourteen studies were included in the final review. Key features of the 14 studies are given in Table 1. Study locations included Belgium, Canada, Israel, Lithuania, and the United States. Studies used a variety of statistical analyses including linear mixed effects models, t-tests, correlational analyses, and analyses of variance to test relationships between nonattachment and other variables. Sample sizes ranged from 10 to 209, with studies 2, 4, and 6 being secondary analyses of studies 1, 3, and 5, respectively. All studies were published in 2014 or later.

### 3.1. Main Findings

This section reports the main findings of the scoping review including an overview, effect sizes and patterns, qualitative insights, cross-sectional analyses, and implications.

### 3.2. Overview of Included Studies

The 14 studies that met the inclusion criteria included 7 randomized controlled trials, 4 cohort studies, and 1 quasi-experimental, 1 cross-sectional, and 1 qualitative study. Sample sizes ranged from 9 to 209 participants (n = 913), and intervention duration ranged from 3 days to 6 months of optional digital platform access. Most interventions adapted established mindfulness-based approaches such as MBSR, MBCT, ACT, or yoga-based formats, which embedded nonattachment-related content including non-judgment, acceptance, letting go, or compassionate awareness. Only two studies explicitly measured nonattachment using NAS [20]. Despite procedural variation, all studies converged on the cultivation of flexible engagement with internal experience, a defining feature of nonattachment.

### 3.3. Effect Sizes and Patterns

Across the seven RCTs, all but one reported statistically significant improvements relative to control conditions. Between-group standardized mean differences (Cohen’s *d*) ranged from approximately 0.6 to 1.1, corresponding to medium-to-large effects on PTSD, anxiety, and depression outcomes. Within-group pre-post improvements were also large (*d* ≈ 0.8–1.2), indicating clinical benefit. In studies directly measuring nonattachment, NAS scores increased by roughly 9%, with higher baseline nonattachment predicting greater reductions in symptom severity (r = 0.55–0.60). A strong dose–response relationship was also observed between session attendance and improvements in both nonattachment and anxiety (r = 0.95, *p* < 0.001).

Programs integrating self-compassion or loving-kindness meditation produced medium standardized effects on affective outcomes (β = −0.10 to −0.30, *p* < 0.01), and interventions emphasizing psychological flexibility or emotional regulation (conceptually aligned with nonattachment) showed consistent gains. One mixed-methods study reported medium-sized changes in affect (*d* = 0.79), and cohort studies focused on acceptance and shame reduction achieved effect sizes of d = 0.77–1.11. Although the smaller yoga-based trials did not yield significant between-group differences (*p* = 0.59), they demonstrated moderate within-group symptom reductions, suggesting physiological stabilization may complement psychological mechanisms of nonattachment.

Nonattachment may have functioned as both a mediator and moderator of therapeutic change. Increases in nonattachment and self-compassion mediated reductions in PTSD and anxiety, while higher baseline nonattachment predicted greater overall improvement, suggesting a resilience-buffering role. Additional mediators included decreased self-criticism, enhanced decentering, and improved emotion regulation. Moderator analyses across studies highlighted the influence of practice dosage, baseline psychological flexibility, and participant readiness. Brief interventions (<4 weeks) generated smaller effects (β = 0.30–0.40), whereas longer MBSR or MBCT-style programs yielded larger and more durable gains (*d* ≥ 0.8).

### 3.4. Qualitative Insights

Qualitative data reinforced quantitative findings, emphasizing experiential themes of staying present, acceptance of adversity, and releasing control. Fifteen military veterans with PTSD completed eight weeks of MBSR and described emergent themes of dealing with the past, staying present, acceptance of adversity, and openness to discomfort. One participant reflected, “There’s a lot of introspection involved, and it’s not necessarily fun sometimes, going back and opening those locked doors, but it helps” [44]. Such narratives exemplify the beginner’s mind orientation, approaching experience with curiosity and compassion, that parallels an attitude of nonattachment. Participants described reduced avoidance and emotional numbing through non-judgmental awareness of distressing emotions, providing interpretive depth to the quantitative findings. Nonattachment may act as an experiential mechanism through which attentional and attitudinal shifts foster trauma integration and emotional recovery.

### 3.5. Cross-Sectional Analyses and Implications

Across studies, descriptive comparison of individual study results (rather than formal meta-analysis) demonstrated effect sizes generally within the medium-to-large range (*d* ≈ 0.8). This may indicate clinically meaningful improvements comparable to or exceeding traditional mindfulness-based interventions for PTSD [45]. Improvements were the strongest for emotion regulation, distress tolerance, and self-compassion, consistent with theoretical models of nonattachment as flexible engagement without clinging or avoidance. Nevertheless, the limited number of trials directly measuring nonattachment and variability in intervention fidelity constrain firm causal inference and therefore findings should be interpreted conservatively.

Across RCTs and cohort studies, reductions in hyperarousal, avoidance, and emotional reactivity were among the most consistent outcomes (*d* ≈ 0.8–1.0). Internet-based MBIs [33,34] and yoga interventions [35,36] improved psychological flexibility and expressive suppression, supporting the interpretation that nonattachment functions by enabling observing without controlling emotional experience. These effects were stronger in interventions with explicit stabilization phases, suggesting that grounding and emotional safety may be prerequisites for nonattachment-based change.

Several studies integrating loving-kindness and self-compassion practices showed large decreases in self-criticism (β ≈ 0.26, *p* < 0.001), shame-driven appraisals and increases in self-compassion (β ≈ −0.10, *p* < 0.01) [37,41]. These results were supported by smaller-scale interventions focusing on acceptance and nonjudgment toward self [42]. This suggests that nonattachment may work partly through softening self-evaluative tendencies and facilitating a more balanced relationship with self-referential thoughts, which echoes tenets of nonattachment-to-self [21]. Collectively, these results support nonattachment’s role as both a process and an outcome of effective mindfulness-based trauma interventions.

## 4. Discussion

This review sought to evaluate MBIs integrating nonattachment elements in populations with PTS symptoms. A total of 14 studies met the inclusion criteria, with the most commonly employed intervention being MBSR (4 studies). Additionally, one study implemented an MBSR/MBCT hybrid intervention. MBSR has no specific module dedicated to nonattachment, yet the underlying principles are applied throughout the program via a focus on observing thoughts, emotions, or sensations without judgement, recognising impermanence, acceptance, releasing automatic or habitual reactivity, and the cultivation of equanimity [15]. All these elements directly link with the concept of nonattachment as it is generally defined [20].

The present scoping review synthesized 14 empirical studies examining mindfulness-based interventions (MBIs) that integrate nonattachment principles for populations experiencing post-traumatic stress. Across these studies, interventions incorporating nonattachment were consistently associated with reductions in PTS symptoms and improvements in mindfulness, psychological flexibility, and self-compassion. Evidence was drawn predominantly from randomized and cohort designs involving adult clinical and refugee populations, with most studies reporting moderate-to-large effects. Collectively, these findings indicate that nonattachment may serve as a cross-cutting mechanism supporting recovery across diverse trauma profiles, offering relevance for both clinical practice and future research on trauma-responsive mindfulness-based approaches.

Three studies used novel MBIs, with the inclusion of elements strongly rooted in nonattachment such as nonjudgement, and a focus on emotional equilibrium. Two studies reported a concurrent focus on elements of Dialectical Behaviour Therapy (DBT) or Acceptance and Commitment Therapy (ACT), reinforcing the significant overlap between these existing intervention modalities. Indeed, consistent with DBT and ACT-based interventions, a key finding of this scoping review was the potential importance of physiological stabilization as part of a phased or multi-modal approach to effective PTS treatment. Another two studies used yoga whilst also being rooted in DBT components, whereby the focus was on physical regulation, self-reflection, and psychological flexibility for PTS symptom mitigation. An interesting observation was that yoga may decrease hyperarousal symptoms, which when not accounted for, may negatively impact intervention outcomes. Thus, a phased or multimodal approach to treatment commencing with stabilization techniques to address hyperarousal may yield more positive intervention outcomes. This supports existing neuroscience research indicating that the prefrontal cortex, hippocampus, and amygdala may be responsible for cognitive and emotional impairment (e.g., hyperarousal), which is commonly associated with PTSD symptoms [46]. Nonattachment practices may support rebalancing within these systems by promoting top-down prefrontal modulation of limbic activity and enhancing hippocampal contextual integration, mechanisms that can reduce hyperarousal and improve emotional regulation [46,47]. These affective–neurocognitive mechanisms likely mirror the patterns of emotional regulation and flexible awareness observed behaviorally, offering a coherent explanatory pathway through which nonattachment may mitigate post-traumatic distress [47].

The primary theme apparent in all studies was nonjudgement. In various ways, all studies spoke to the importance of this element for beneficial outcomes such as reduced avoidance, increased acceptance, and an understanding of thoughts and emotions as transient and impermanent. An interesting pattern across the data was the focus on nonjudgement as a potential mechanism by which participants might practice presence. Existing MBIs or MBI-adjunctive therapies acknowledge the importance of this element through a variety of targeted approaches to judgement reduction. For example, MBSR dedicates program time to learning how to respond with mindful intention and observation of experience without judgement. DBT [48,49] teaches ‘wise-mind’ to practice a non-judgemental stance toward self and others. ACT [16] offers training in observation of thoughts and sensations without judgement. In Compassion Focused Therapy [50] focus is placed on learning to embrace vulnerability or imperfections without judgement (see Tremblay et al. [12] for a detailed review of how such therapeutic modalities integrate nonattachment principles). Thus, building nonjudgement may facilitate a higher tolerance for being in the moment, which may allow one to foster attentional control through a reduced need to avoid or escape to the past (rumination) or future (anxiety). Stated differently, nonjudgement may be a critical contributor to the ability to observe without a need to alter or avoid, which is a core tenet of nonattachment.

Although one study [37] found that reduction in self-criticism mediated the effects of the intervention more than self-compassion, the importance of self-compassion was evident throughout, whereby self-compassion was usually facilitated by meditations or psychoeducation geared toward loving kindness, a reduction in shame, and/or increasing capacity for acceptance. Compassion focused inwardly more so than outwardly appeared to foster salutary outcomes which closely resemble those of nonattachment. For example, nonattachment scale scores have been correlated with reductions in PTSD symptom severity, anxiety sensitivity, rejection sensitivity, and changes in empathic concern and personal distress aspects of empathy [28]. CFT focuses on these areas through reduction in shame, a more open and non-judgemental stance toward self and others, and the practice of loving-kindness through both meditation and psychoeducation [50]. This may also be cultivated in MBSR [15] through focus on kindness and understanding to self and others. Similarly, MBCT [51] targets self-compassion through training to release judgement and reactivity and accept experiences as they are. As with nonjudgement, DBT [48,49] uses cultivation of ‘wise-mind’ to improve self-compassion.

A tertiary theme which appeared in most studies was an element of participant influence or control. Participants were often given options on how to engage with the intervention, including exerting a limited amount of control on variables such as length of time spent in meditation, option to speak or not during group sessions, focus areas, and autonomy to emphasize individually preferred practices such as yoga, breathwork, or body-scan meditations. Where participant influence was designed and offered, discussions cited this element as a potentially valuable contributor to the effectiveness of the intervention.

Taken together, these studies offer evidence indicating a role for nonattachment-based principles embedded in MBIs for treating PTS. All interventions included elements of compassion, which is important in reducing attachment to self-criticism, judgement, and negative self-identity [50]. Several studies also focused on nonreactivity; a detachment from habitual or automatic responses which should be replaced with learning how to respond mindfully and intentionally. This may be facilitated by psychoeducation related to the transient nature of thoughts and emotions, which in turn may promote a higher tolerance for being present with uncomfortable feelings. This connects strongly with nonattachment in which intentional viewing of thoughts and experiences as external, transient, objectively neutral, and finite are core tenets [17]. It may therefore be that cultivating nonattachment is the key to a neutral or open recognition that a thought or emotion is simply that, versus a judgemental or emotionally driven response. Thoughts or emotions can be seen, felt, and released. This may be much easier said than done, especially in relation to PTS where the internalized experience of the event can feel present, current, and deeply triggering. However, with stabilization and intentional practice, attentional and attitudinal shifts may be possible.

Most interventions offered psychoeducation and independent practice exercises to participants. Participant flexibility or autonomy built into the structure may be particularly appropriate for people suffering with PTS symptoms because working at the primary direction of an intervention administrator or mental health practitioner may risk compounding feelings of helplessness or loss of control. Indeed, one of the studies reviewed found that patients who engaged in shared decision making for their treatment planning were highly (90%) likely to report feeling confident about moving forward with the plans created [52]. Autonomy or person-centred input may therefore be an important contributor to intervention success.

### 4.1. Limitations

This review may have been limited by several factors including restricting the scope to English language papers. Additionally, excluding articles not published in peer-reviewed journals may have meant that relevant data was overlooked. Also, the eligibility criteria underlying the study selection process may have resulted in the exclusion of studies that tap into constructs that are related but distinct from nonattachment. Additionally, despite methodological steps taken to reduce selection and interpretation bias, it is not possible to completely rule out researcher subjectivity as part of the identification and appraisal of studies. Finally, because most included studies used varied interventions and focused on specific populations, the results of this review may not be generalisable to all populations with PTS, or to all forms of MBIs.

### 4.2. Implications and Recommendations

Current MBIs often contain dedicated program time and attention to increasing acceptance, which is a critical element of nonattachment; mindful awareness of what is without the need to alter or assign judgement. This may be particularly relevant for PTS application as research shows that self-judgement, self-criticism, and self-blame can be particularly salient and painful outcomes of PTS [2]. Increased baseline scores in these areas could indicate a higher likelihood of PTS onset. Therefore, training in the intentional direction of attention, coupled with a foundation of psychoeducation dedicated to relevant principles may yield beneficial outcomes associated with accepting life events as they are instead of responding to urges to alter or control them. This may not seem to differ substantially from traditional MBIs; however, none of the contemplative modalities that have been researched to date exclusively or primarily employ nonattachment techniques. Introducing nonattachment-based psychoeducation, coupled with teaching conscious attention and nonattachment practices, may thus be advisable. Additionally, certain populations may benefit from a more multi-modal approach to treatment whereby stabilization and normalizations are targeted before anything else. This would likely be particularly useful for populations suffering from symptoms of PTS where the event can still feel present, tripping the neural circuitry required for onset of fight or flight, or amygdala activation [46,47,53]. While some existing MBI modalities may address this step (e.g., DBT begins by targeting life-threatening behaviours and achieving behavioural stability), most do not do so explicitly. That is, existing modalities may accomplish comparable goals to stabilization, but without participant understanding, agreement and input, their efficacy may be reduced [52]. While the current evidence base demonstrates promising effects of nonattachment-informed interventions for trauma, most studies were characterized by relatively small sample sizes and limited demographic diversity.

Future research should prioritize larger randomized controlled trials across multiple settings to evaluate the scalability and generalizability of these findings across cultures, age groups, and trauma contexts. Given that nonattachment emphasizes flexible engagement with experience, it may be particularly adaptable to diverse sociocultural frameworks; however, empirical testing of this adaptability remains limited. Future research could explore how nonattachment-based modules may be integrated into digital delivery formats, community-based trauma programs, and culturally tailored mindfulness curricula, assessing both accessibility and fidelity. Such work would clarify whether the observed benefits extend to under-represented populations, including men, non-Western samples, and individuals with complex trauma or comorbid conditions

## 5. Conclusions

This scoping review sought to fill a gap in existing literature regarding which elements of existing MBIs for trauma populations are nonattachment-based. This review contributes the first synthesis of data dedicated to nonattachment within existing MBIs, and suggests that within the context of PTS, incorporation of nonattachment-based principles or practices may have therapeutic modality. Evidence within this review lends empirical support to the theoretical notion of utility of nonattachment for use in PTS contexts. Although nonattachment has been demonstrated as a distinguishable construct from mindfulness, it faces an application hurdle in the lack of specific and applied guidelines for its use in trauma treatment [54]. Nonattachment-based elements most likely to be playing a role in effective PTS application include a reduction in judgement, self-criticism and rumination, and increased capacity for acceptance and compassion. Additionally, targeted reconstruction of an understanding of self as more than thoughts, emotions, and sensations (nonattachment-to-self) was noted as an important and useful potential contributor to mitigation of PTS symptoms. These areas of focus align with the definition of nonattachment as an attitude or state of being in which one can accept and also let go of life events as they are [20]. The nonattached individual can attend to the present without a need to assign positivity or negativity through judgement [12], which may help foster an attitude of compassion toward self and others. Key findings included the identification of a phased approach as a potentially valuable step in the efficacy of an intervention, with stabilization being a preliminary step which may act to facilitate the effectiveness of the subsequent phases. Another key finding was the importance of participant input, which was a factor in several interventions in the studies reviewed. This provides support for future interventions including participant input as a feature of design.

## Figures and Tables

**Table 1:** Key Aspects of Included Studies.

Study No. (Design)	Article Identifiers and SQAC Score *	Intervention Type	Population Description and Location	Control Condition (If Applicable)	Key Outcomes
1 (Randomised Controlled Trial/RCT)	Effects of a MBI on Self-Compassion and psychological Health Among Young Adults with a History of Childhood Maltreatment [32]. 80%	MBSR, 8 weeks × 2.5 h plus 1 full day/week	n = 38, age 22–29, individuals who were maltreated in childhood. USA.	Waitlist (n = 18)	MBSR can improve self-compassion and psychological health
2 (RCT)	Nonattachment predicts Empathy, Rejection Sensitivity, and Symptom Reduction after a MBI Among Young Adults with a History of Childhood Maltreatment [28]. 80%	MBSR, 8 weeks × 2.5 h plus 1 full day/week	n = 38, age 22–29, individuals who were maltreated in childhood. USA.	Waitlist (n = 18)	MBSR can improve mindfulness, Nonattachment and empathy which can impact interpersonal distress and rejection sensitivity
3 (RCT)	Effects of a MBI on ICD-11 PTSD and CPTSD Symptoms: A Pilot Randomized Controlled Trial [33]. 88%	Novel MBI based on principles of awareness and nonjudgement of physical senses, thoughts, and emotions. 8 weeks of 1 meditation/day lasting 2–7 min	n = 70, age 20–35 meeting criteria for PTSD, CPTSD or Disturbances in self-organization. Lithuania.	Waitlist (n = 39). Could access the program after 5 months	MBI can reduce CPTSD symptoms and have positive effects on overall mental health. Internet-based interventions seem effective for certain types of trauma care
4 (RCT)	The effects of Online MBI on PTSD and CPTSD Symptoms: A Randomized Controlled Trial with 3-Month Follow-Up [34]. 88%	Novel MBI based on principles of awareness and nonjudgement of physical senses, thoughts, and emotions. 8 weeks of 1 meditation/day lasting 2–7 min	n = 70, age 20–35 meeting criteria for PTSD, CPTSD or Disturbances in self-organization. Lithuania	Waitlist (n = 39). Could access the program after 5 months	Effects of MBI for PTSD and CPTSD symptoms retained over time (3 months)
5 (RCT)	A Pilot Study of a RCT of Yoga as an Intervention for PTSD Symptoms in Women [35]. 87.5%	Yoga-based intervention. 12 weeks with 1 × 75 min/week or 6 weeks with 2 × 75 min	n = 38, mean age = 44 veteran and civilian adult females with full or subthreshold PTSD symptoms. USA.	Waitlist (n = 18) completed same weekly questionnaires as exp condition in weekly group meetings	Yoga intervention yielded decreases in reexperiencing and hyperarousal. Intervention is tolerable and may be an effective adjunctive intervention for this population.
6 (RCT)	Examining Mechanisms of Change in a Yoga Intervention for Women: The Influence of Mindfulness, Psychological Flexibility, and Emotion Regulation on PTSD symptoms [36]. 87.5%	Yoga-based intervention. 12 weeks with 1 × 75 min/week or 6 weeks with 2 × 75 min	n = 38, mean age = 44 veteran and civilian adult females with full or subthreshold PTSD symptoms. USA.	Waitlist (n = 18) completed same weekly questionnaires as exp condition in weekly group meetings	Yoga may reduce expressive suppression and improve PTSD symptoms. Psychological flexibility increased for control but not exp group, counter to predictions.
7 (RCT)	Candidate Mechanisms of Action of Mindfulness-Based Trauma Recovery for Refugees: Self-Compassion and Self-Criticism [37]. 83%	Mindfulness-based Trauma Recovery for Refugees. 9 weeks × 2.5 h	n = 158, age = 20–48, traumatized East-African asylum-seekers residing in Israel.	Waitlist (ratio of 3:2 exp to control) who were able to access intervention after exp completion	Intervention yielded improvements in self-compassion and reductions in self-criticism. Findings indicated importance of self-referentiality as a target mechanism in MBIs and trauma recovery.
8 (Quasi Experimental)	Promoting Attachment-Related Mindfulness and Compassion: a Wait-List Controlled Study of Women Who Were Mistreated During Childhood [38]. 75%	REAC2H program focused on mind, thoughts, and emotions over a 3-day × 8 h intensive	n = 17, age 18–80 participants who self-reported childhood maltreatment.	Waitlist n = 22	Significant improvements in rumination, emotion suppression, emotion regulation, clarity of emotions, and mindfulness.
9 (Cross-Sectional)	Mindfulness and Metta-Based Trauma Therapy (MMTT): Initial Development and Proof-of-Concept of an Internet Resource [39]. 92.5%	Novel Metta based trauma therapy where one engagement constituted participation with unlimited access for 6 months	n = 177, age 18–75 participants who self-reported suffering from PTSD symptoms using PCL-5 assessment. Canada.	None	Participants reported utility of intervention as credible and helpful for improving self-regulation, wellbeing, and mitigating PTSD, anxiety, depression, and dissociation. Participants with higher PTSD symptoms enjoyed metta meditations less than those with less intense symptoms
10 (Cohort-mixed method)	A MBI for Unaccompanied Refugee Minors: A Pilot Study with Mixed Methods Evaluation [40]. 81.8%	MBSR-MBCT Hybrid with 8 weeks × 90 min	n = 13 aged 13–18, who were unaccompanied minor refugees. Belgium.	None	MBI may decrease negative and increase positive affect and reduce symptoms of depression. Mindfulness exercises may be used as a coping strategy
11 (Cohort)	MBSR for PTS Symptoms: Building Acceptance and Decreasing Shame [41]. 90.9%	MBSR with 8 sessions said to have followed MBSR guidelines, but unspecified in duration	n = 9, average age = 44, adults who reported trauma exposure, PTS, or depression. USA.	None	PTS, depression, and shame-based trauma appraisals decreased. Acceptance of emotional experiences increased. Reducing shame and increasing acceptance may be important in trauma recovery
12 (Cohort)	Decreasing Perceived and Academic Stress Through Emotion Regulation and Nonjudging with Trauma-Exposed College Students [42]. 88%	Novel MBI focused on enhancing emotional regulation and nonjudgement to reduce stress with 3 weeks × 10 min meditations (1/week)	n = 209, age 18–42 undergraduate students who reported trauma exposure. USA.	No description	A brief MBI can reduce academic and perceived stress through emotional regulation and increasing nonjudgement. Perceived stress was only reduced in participants with subthreshold PTSD
13 (Cohort)	Testing the Acceptability and Initial Efficacy of a Smart-phone App Mindfulness Intervention for College Student Veterans with PTSD [43]. 86%	Novel MBI with elements of ACT for 4 weeks with at least one meditation per day (6–19 min long) plus weekly phone check-ins	n = 23, age 23–43 student veterans	None	App delivery was favourably rated. Improvements noted in resilience, mindfulness, PTSD symptoms, experiential avoidance, and rumination
14 (Qualitative, using semi-structured interviews)	Mindfulness-Based Process of Healing for Veterans with PTSD [44]. 80%	MBSR with 8 weeks × 2.5 h and 1 full day/week.	n = 15, age unspecified, veterans with a PTSD diagnosis. USA.	None	Six core aspects of MBSR experience were identified, including: dealing with past, staying in present, acceptance of adversity, breathing through stress, relaxation, and openness to self and others. Introspection and curiosity may have been activated by MBSR participation

* The primary study quality issue apparent to a full or partial extent in all of the included quantitative studies was a lack of control for potentially confounding variables, such as comorbidities or adjunct therapies. Limited sample size in many studies may have influenced the validity and reliability of results. Additionally, control conditions received limited mention beyond a label of waitlist in most studies, and no studies were designed for blinding of both participants and investigators. However, controlling for confounds may be difficult given the complex nature of PTS symptoms, which are frequently comorbid with those of other diagnoses, particularly anxiety and depression [1,41]. Nevertheless, the included studies generally described objectives, designs, and measures well. Additionally, all quantitative studies reviewed provided some estimate of variance for main results. Conclusions in all studies were generally well supported by the results. SQAC: Standard Quality Assessment Criteria; RCT: Randomized Controlled Trial; MBI: Mindfulness Based Intervention; MBSR: Mindfulness Based Stress Redcution; ICD-11: International Classification of Diseased, version 11; PTSD: Post Traumatic Stress Disorder; CPTSD: Complex Post Traumatic Stress Disorder; REAC2H: Restoring Embodied Awareness, Compassionate Connection, and Hope; PCL: Posttraumatic Stress Disorder Checklist; MBSR-MBCT: Mindfulness Based Stress Reduction–Mindfulness Based Cognitive Therapy; PTS: Post Traumatic Stress; ACT: Acceptance and Commitment Therapy.

## Data Availability

No new data were created or analyzed in this study. Data sharing is not applicable to this article.

## Supplementary Materials

The following supporting information can be downloaded at: https://www.mdpi.com/article/10.3390/jpm15120614/s1. Table S1: PRISMA Checklist.

## Abbreviations

The following abbreviations are used in this manuscript:

(C)PTS (D)	(Complex) Post-Traumatic Stress (Disorder)
SAMHSA	Substance Abuse and Mental Health Administration
CBT	Cognitive Behavioural Therapy
MBI	Mindfulness-based Intervention
ACT	Acceptance and Commitment Therapy
NTS	Nonattachment to Self
SQAC	Standard Quality Assessment Criteria
MBSR	Mindfulness-Based Stress Reduction
DBT	Dialectical Behavioural Therapy
CFT	Compassion-Focused Therapy
